# The signs of Antarctic ozone hole recovery

**DOI:** 10.1038/s41598-017-00722-7

**Published:** 2017-04-03

**Authors:** Jayanarayanan Kuttippurath, Prijitha J. Nair

**Affiliations:** 1 CORAL, Indian Institute of Technology Kharagpur, Kharagpur, 721302 India; 2 LATMOS/CNRS, UPMC University of Paris 06, Paris, France; 3 Centre for Earth Science Studies, Thiruvananthapuram, India

## Abstract

Absorption of solar radiation by stratospheric ozone affects atmospheric dynamics and chemistry, and sustains life on Earth by preventing harmful radiation from reaching the surface. Significant ozone losses due to increases in the abundances of ozone depleting substances (ODSs) were first observed in Antarctica in the 1980s. Losses deepened in following years but became nearly flat by around 2000, reflecting changes in global ODS emissions. Here we show robust evidence that Antarctic ozone has started to recover in both spring and summer, with a recovery signal identified in springtime ozone profile and total column measurements at 99% confidence for the first time. Continuing recovery is expected to impact the future climate of that region. Our results demonstrate that the Montreal Protocol has indeed begun to save the Antarctic ozone layer.

## Introduction

Since the discovery of the Antarctic ozone hole in the mid-1980s, a great deal of scientific attention has focussed on understanding the causes and evolution of the ozone loss in both Polar Regions^1–3^, as the ozone layer is essential to support life on the Earth by preventing harmful solar ultraviolet radiation from reaching the surface. The ozone loss in the Antarctic has been particularly severe owing to the special meteorological condition and springtime chemistry there^4, 5^. Antarctic spring ozone losses levelled off by the late 1990s^5^, but substantial depletion continues to occur in Antarctica each spring to date^6^. Recent studies indicate that the Antarctic ozone loss has affected the temperature^7^, wind patterns^8^, precipitation^9^, ocean^10^, ecosystems^11^ and circulation patterns^12^ in the southern hemisphere.

Analyses of springtime total column ozone (TCO) measurements in the Antarctic have suggested significant positive trends towards its recovery^13, 14^ as a result of actions taken under the Montreal Protocol to control the emissions of man-made ozone depleting substances (ODSs). One of the most recent studies on Antarctic ozone trends shows that there is a significant positive trend in the Antarctic ozone in spring^15^. Since the Antarctic ozone loss is primarily confined to the polar vortex in spring, a vortex based trend analysis is necessary to unveil the emerging healing sings of the ozone and is not performed yet. Therefore, in this work we analyse the vortex-oriented ozone trends in Antarctica using ozonesonde profiles and satellite TCO measurements by applying a different trend detection method.

Ensuring that ozone changes are indeed due to chemistry rather than dynamical variability is a challenging task. Evident shifts in dynamical processes in the spring impeded clear attribution of chemical effects in a study of detailed, height-resolved ozonesonde measurements at South Pole and Neumayer stations^16^. Recent satellite measurements also show changes in ozone loss rates that are difficult to distinguish from an observed springtime dynamical shift in Antarctica in the past decade^17, 18^. The reported springtime dynamical changes involve shifts in the location of the vortex and longitude of planetary wave structure^15^, as well as in the character of spring stratospheric minor warmings^16^. A key aspect of this study is the consideration of summer season ozone changes along with those for spring. While ozone losses in summer are smaller than those in spring, the dynamical conditions in summer are far more quiescent, which greatly facilitates interpretation. Impacts of ozone depletion on tropospheric climate are greatest in the summer^12^, further motivating interest in that season.

One of the important restrictions on long-term trend analysis of Antarctic ozone is limited data to diagnose the vertical trends. The lack of continuous measurements from a single satellite instrument and poor coverage of polar latitudes complicate trend analysis^19, 20^. In order to overcome these limitations, we use ozonesonde measurements from Antarctica^3, 4, 6^, which have continuous long-term measurements with very high vertical resolution over about 10–35 km. There are seven regular ozone sounding stations in Antarctica used here: Davis, Georg-Foster, Maitri, Marambio, McMurdo, Mirny, Neumayer, South Pole and Syowa (see co-ordinates, measurement details and data analysis in Methods). This is the first study that uses measurements exclusively inside the Antarctic vortex for long-term ozone profile trend analysis, by sorting with respect to a vortex edge criteria (i.e. inside the 65° S Equivalent latitude – EqL or Nash *et al*. criterion)^21^ at each altitude from 250 to 950 K (~7–35 km, see Supplementary Figure S1).

## Drivers of ozone change and past trends

**Figure 1:**
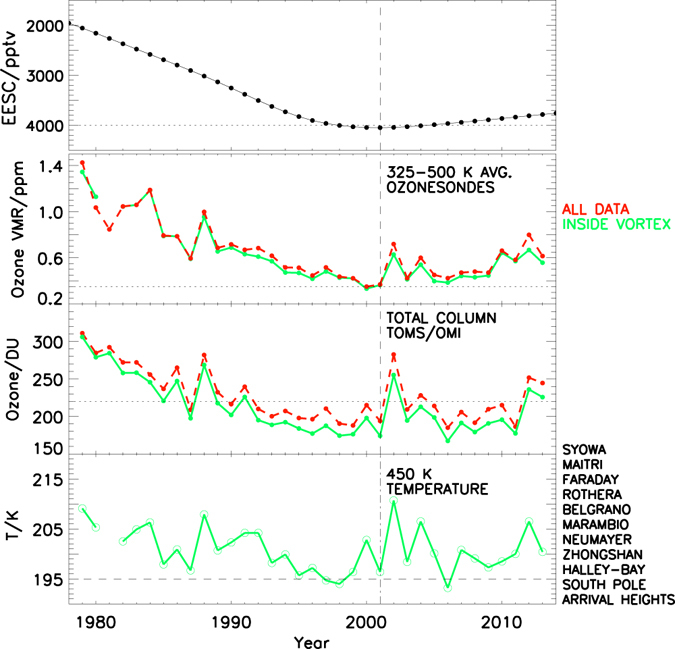
Time evolution of stratospheric halogens, ozone and temperature: Top: The temporal evolution of equivalent effective stratospheric chlorine (EESC) in the stratosphere in the inverted scale (with respect to the World Meteorological Organisation A1–2010 scenario). The horizontal line indicates 4000 pptv. Second from Top: Time evolution of the September, October and November (SON, spring) averaged ozone volume mixing ratios (VMRs) over the altitude range 325–500 K (~10–20 km) from ozonesonde measurements in the Antarctic. The horizontal line indicates 0.4 ppmv. Third from Top. The time evolution of the SON averaged total column ozone (TCO) measurements at selected Antarctic stations, as listed in the figure legend. The TCO measurements are taken by the Total Column Ozone Mapping Spectrometer (TOMS, 1979–2003) and Ozone Monitoring Instrument (OMI, 2004–2013). The horizontal line indicates 220 DU, the Antarctic ozone hole criterion. Bottom: The vortex-averaged temperature estimated at the ozonesonde stations in the Antarctic vortex in spring (SON). The horizontal dotted lines represent 195 K. The red curve shows all measurements without any vortex consideration (ALL DATA) and the green curve represents the measurements sorted inside the vortex with ≥65° S EqL criterion (INSIDE VORTEX). The vertical dashed line at 2001 in all plots represents the turnaround year of EESC.

The September, October and November (SON) average ozone is used for spring season trend analyses. Since the average breakup date of the Antarctic vortex is around day 334 (November 30)^22^ and we consider the measurements inside the vortex, this represents the maximum ozone loss period^13, 14^. Figure 1 shows that the measured ozone abundances averaged over the region of extreme depletion from about 10 to 20 km (~325–500 K) show a slight increase in 2001–2013 relative to the average over 1979–2001, in broad agreement with the decline in Equivalent Effective Stratospheric Chlorine (EESC) in that period. Similar behaviour is obtained in the total column (Fig. 1) and over the range of depleted altitudes from 9 to 28 km (300–700 K, see Figure S1 [right]). Note that the vortex-averaged ozone in each period is illustrated in Figure S1 (left). Therefore, it shows larger loss during the peak halogen period in 2001–2013. Since a number of factors can influence the long-term changes in ozone (e.g. Supplementary Figure S2) abundances at each latitude and altitude^13, 14^, a simple temporal average or linear trend analysis is not sufficient to identify chemical changes. Hence the multivariate piece-wise linear trend (PWLT) regression is applied^23^. Here time is a proxy and the trends are estimated before and after the year of EESC maximum in the polar stratosphere (i.e. 2001)^24^. The regression procedure incorporates all of the key proxies known to influence the long-term evolution of ozone in the Antarctic stratosphere^14^, and below we examine in detail the sensitivity of our findings to which proxies are included or excluded. The proxy data consist of the eddy heat flux averaged over 45°–75° S at 100 hPa to represent the effect of Brewer-Dobson circulation on ozone change^25^, the Antarctic Oscillation (AAO) index^26^ and the Quasi-Biennial Oscillation (QBO)^27^ at 40 hPa to represent the secondary circulation or local meteorological patterns influencing the ozone distribution, Solar Flux (SF) at 10.7 cm wavelength to account for the changes in ozone due to solar activity^28^, and aerosol data, to represent the enhanced loss induced by the El Chichon (1982) and Mount Pinatubo (1991) volcanic aerosols^29, 30^. Then, the change in ozone is given as:

$$\begin{array}{rcl}{{\rm{O}}}_{3}({\rm{t}}) & = & {\rm{Constant}}+{{\rm{C}}}_{1}{{\rm{t}}}_{1}+{{\rm{C}}}_{2}{{\rm{t}}}_{2}+{{\rm{C}}}_{3}{\rm{Solar}}\,{\rm{Flux}}({\rm{t}})\times {\rm{QBO}}({\rm{t}})\\  &  & +\,{{\rm{C}}}_{4}{\rm{Heat}}\,{\rm{Flux}}({\rm{t}})+{{\rm{C}}}_{5}{\rm{Aerosol}}({\rm{t}})+{{\rm{C}}}_{6}{\rm{AAO}}({\rm{t}})+{\rm{R}}({\rm{t}})\end{array}$$

where t is time period from 1979 to 2013, t_1_ is the number of years from 1979 to 2013, t_2_ is the number of years from 2001 to 2013, C_1_t_1_ is the linear trend, C_2_t_2_ is the change in trend, R is the residual and C1 to C_6_ are the regression coefficients of the respective proxies. The regression yields the linear change in ozone after removing the influence of all proxies. The error of the regression coefficient is calculated using the generalised least-squares method^31^ and by considering the autocorrelation of residuals with one year lag. Note that the difference between the measurement and regressed output (i.e. the residual) also affects the estimated uncertainties for the trends, i.e. if the fit is not good, the uncertainty of the estimated trends will be correspondingly large too.

**Figure 2:**
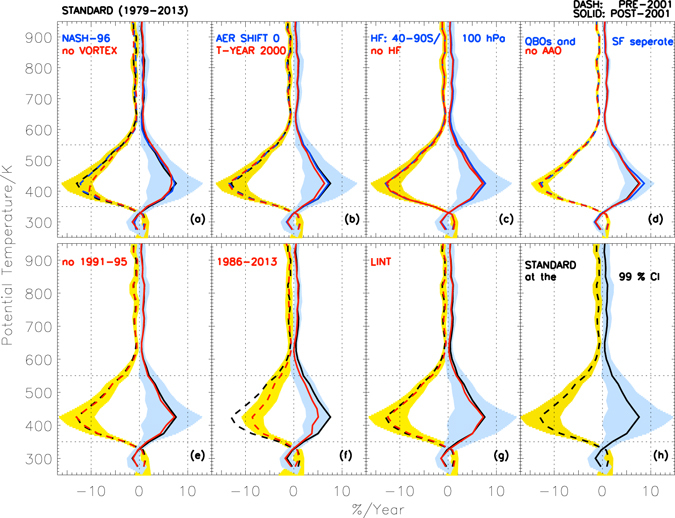
Vertical structure of Antarctic ozone recovery: (**a**) The ozone trends estimated from ozonesonde data in the Antarctic vortex in spring (SON) with ≥65° S EqL criterion (“standard”, black cures), Nash *et al*.^20^ vortex criterion (“Nash-96”, blue curves) and without considering the vortex boundaries (“no VORTEX”, red curves) for the 1979–2001 (dash) and 2001–2013 (solid) periods. The break year 2001 corresponds to the year of maximum stratospheric chlorine in the Polar Regions. **(b)** Trends estimated with various input scenarios, where the blue curves (“AER SHIFT 0”) represent those found without the aerosol time lag and the red curves (“T–YEAR 2000”) represent the analysis performed by changing the break-year to 2000. **(c)** Trends estimated with a different latitude range for the heat flux data (blue curves) and without the heat flux term entirely are also shown (red curves). In all plots (**a**–**c**) the standard estimate (black curves) and its uncertainty at the 95% level (shaded areas) are shown. **(d)** Trend computed without the Antarctic Oscillation (AAO) term and shaded areas represent its uncertainty at the 95% level (red curves). Trend computed using the solar flux (SF) and Quasi-biennial Oscillation (QBO) terms separately (blue curves) instead of coupling SF and QBO as for the standard scenario. **(e)** Trend computed without the 1991–1995 data (red curves) and shaded areas represent its uncertainty at the 95% level. **(f)** Trend estimated without the 1979–1985 data (red curves) and shaded areas represent its uncertainty at the 95% level. **(g)** Trend estimated without using any regression terms, but with the linear terms (“LINT”); shaded areas represent its uncertainty at the 95% level. **(h)** Standard scenario with its uncertainty at the 99% level. In all plots (**a**–**h**) the horizontal dotted lines represent 350 K (~12 km) and 550 K (~22 km) altitudes and vertical dotted lines represent −10%/year and 10%/year.

The ozone trends computed after removing the influence of proxies for the pre-EESC maximum period are shown in Fig. 2a (marked as “standard” in dashed black curve). In 1979–2001, negative trends are found throughout the region from about 12 to 25 km (~350–600 K), with −3%/year at 350 K, which gradually increases to −13%/year at 425 K (~15 km) at the centre of the saturation layer (as depicted in Supplementary Figure S4c). The trends gradually reduce to −3%/year at 550 K and remain at about −1%/year above that altitude. An analysis performed in absolute units (parts per million [ppm] per year), instead of per cent per year shows depletion of about −0.06 to −0.075 ppm/year at 400–575 K (~13–23 km) and about −0.05 ppm/year above 700 K (~28 km, see Figure S4b). Trends are statistically different from zero at the 95% confidence interval above 330 K (~11 km). A recent study using ground-based UV reflectance (Umkehr) measurements capable of detecting ozone averaged over 5–10 km thick layers in the Antarctic showed similar values of about −2 to −3%/year at the lower stratospheric Umkehr layers^32^.

## Vertical profile of ozone recovery

The trends found for the post EESC-maximum period (2001–2013) suggest significant increases in spring ozone indicative of recovery throughout the region from 325 K (~10 km) up to 550 K (Fig. 2a, black solid curve), i.e. about +2%/year at 350 K, gradually increasing to +8%/year at 425 K, and reducing gradually to +2%/year at 550 K. The analysis in absolute units shows a corresponding increase of +0.03 to +0.04 ppm/year at 425–575 K and around +0.03 ppm/year above 700 K (see Figure S4b). Both analyses show trends statistically significantly different from zero at the 95% confidence interval at 350–550 K (~12–22 km), i.e. at the core of the Antarctic ozone hole or the ozone loss saturation layer (as shown in Figure S4c). Furthermore, the trends are also significant at the altitude ranges 560–580 K (~23–24 km) and 650–710 K (~26–28 km). In general, the uncertainty of the estimated trends is larger for the latter period (where there are fewer number of years), as the trends and their uncertainty estimates by PWLT depend also on the number of years.

Space-based TCO measurements at the locations of Arrival heights, Belgrano, Faraday, Halley, Marambio, Neumayer, Rothera, Zhongshan, South Pole and Syowa are presented for comparison in Table 1. Positive trends at the 95% confidence level have already been reported post-2000^14^. Here we perform the vortex-based analyses for the 1979–2013 period, and we also present a separate analysis without the vortex discrimination (Fig. 1, bottom). The TCO trends inside the vortex (≥65° S EqL) show about −2.34 ± 0.40%/year (or −4.99 ± 0.86 DU/year) over 1979–2001 and +1.72 ± 0.81%/year (or +3.68 ± 1.73 DU/year) for the 2001–2013 period. An analysis is also performed without the 1991–1995 period affected by the heterogeneous ozone loss on Mount Pinatubo volcanic aerosols. The trends are slightly smaller and the uncertainty increases noticeably when the 1991–1995 data are not considered, while the estimates are about 0.1–0.2%/year smaller for all measurements without the vortex condition. Table 1 shows that these results are robust to choices regarding proxies. Very similar results are obtained whether or not the influence of planetary wave drive or heat flux is included in the analysis (Table 1, third panel from top). Furthermore, since the long-term changes in the AAO are thought to be linked to ozone changes^12, 26^, we also computed trends when this proxy is not included. The resulting analysis again gives trends similar to the standard scenario (Table 1, bottom). Nevertheless, the trends are significant at the 95% level for observations both inside and outside the vortex.

**Table 1:** Total Column Ozone trends in Antarctic Spring: The piece-wise linear trends (PWLT) estimated from satellite total column ozone (TCO) measurements averaged over September, October and November (SON, spring) from selected Antarctic stations.

	1979–2001%/yr	2001–2013%/yr
**All data**		
EqL	−2.34 ± 0.40	+1.72 ± 0.81
Edg	−2.34 ± 0.40	+1.80 ± 0.80
All data	−2.03 ± 0.39	+1.50 ± 0.79
**Without the 1991–1995 data**		
EqL	−2.17 ± 0.46	+1.39 ± 0.89
Edg	−2.16 ± 0.46	+1.48 ± 0.91
All data	−1.85 ± 0.45	+1.16 ± 0.89
**Without Heat Flux**		
EqL	−2.10 ± 0.51	+1.34 ± 1.03
Edg	−2.12 ± 0.48	+1.46 ± 0.99
All data	−1.79 ± 0.50	+1.12 ± 1.02
**Without Antarctic Oscillation (AAO)**		
EqL	−2.40 ± 0.47	+1.80 ± 0.95
Edg	−2.39 ± 0.47	+1.87 ± 0.95
All data	−2.09 ± 0.41	+1.57 ± 0.83

The error bars represent the 95% confidence interval. The measurements averaged over the vortex criteria ≥ 65° S EqL are represented by *EqL*, the measurements averaged over the Nash *et al*.^20^ vortex criteria are represented by *Edg*, and the averages of data poleward of 65° S without considering vortex definitions are represented by *All data*.

## Strength of the recovery signal

To further assess the robustness of the spring season recovery trends estimated over 2001–2013, we performed several additional tests of different input data scenarios to represent the uncertainty of the regression terms (e.g. changes in vortex edge definition, turnaround year, exemption of volcanic aerosol years, etc.). For instance, we used the vortex edge with respect to the potential vorticity (PV) values, instead of the ≥65° S EqL criterion, at each altitude as proposed by Nash *et al*.^21^; various vortex criteria yielded very similar results (Fig. 2a). When the analyses were performed without any vortex criterion at all, the trends were smaller and uncertainty increased, with differences of −1 to −3%/year at 380–500 K (~13–20 km) in 1979–2001 and about −1%/year at 390–410 K (around 14 km) in 2001–2013; nonetheless, trends remained significantly different from zero.

In the standard regression the aerosol data have a time shift of +6 months, which optimized the performance of the regression^14^. The estimated trends, however, did not vary significantly from the standard scenario when no time lag was applied in the analysis (Fig. 2b, “AER SHIFT 0”). Similarly, an analysis with a turnaround year in 2000, instead of 2001 was tested, since the peak in EESC was observed around April 2000. The resulting estimates show more or less the same trends in 1979–2000, but about 0.5–1.5%/year smaller values at 375–425 K (~14–15 km) in 2000–2013 compared to the standard scenario.

It is well known that the temperature controls the polar stratospheric cloud (PSC) formation, chlorine and bromine activation, and hence, the springtime ozone depletion^1, 5^. For instance, Fig. 1 bottom panel shows the time series of average temperature form all Antarctic stations and it illustrates an excellent correspondence with the variations in both total and partial column ozone measurements. Therefore, to understand the impact of year-to-year variability of temperature and Antarctic meteorology on the recovery trends, we computed trends with different heat flux scenarios. We found that changing the latitudinal average (40°–90° S) or entirely omitting the heat flux in the regression did not yield significant changes in the estimated trends within uncertainties (Fig. 2c). This is a key finding of this paper since it indicates clear ozone recovery even without subtracting the variability induced by dynamics. However, ozone and heat flux are coupled, and a linear relationship between inter-annual variability in polar cap (50°–90°) ozone and extra-tropical heat flux is well established^33, 34^. This ozone and heat flux relationship also suggests a feedback mechanism and hence, we have subtracted the heat flux in the regression to diagnose the dynamical contribution to ozone evolution. Likewise, a test is also performed by subtracting the influence of AAO term in the analysis (Fig. 2d). The resulting estimates showed equivalent trends with smaller uncertainty ranges in both periods. During the high phase of AAO, the Lagrangian mean circulation induced transport of ozone from low latitudes to high latitudes is strongly reduced. This is due to atmospheric wave activity. The opposite scenario occurs during the low phase of AAO. Therefore, the results indicate the effect of dynamics on ozone distribution with altitude. The QBO and SF are combined in the regression procedure to best account for coupling via polar vortex temperature as in prior studies^28^, but the trend values are nearly identical if the two terms are regressed separately (Fig. 2d, blue curves). The uncertainty range in the latter case, however, was smaller than that of the standard scenario and was similar to those displayed in the figure for the “no AAO” analysis.

The analysis performed without the volcanic aerosol affected 1991–1995 data shows slightly smaller values (maximum of about 0.5%/year) in both periods (Fig. 2e and Table 1). However, the uncertainty range (shaded areas) is larger than the standard scenario. The sensitivity of the trends was also checked with an analysis that excludes the 1979–1985 data, as there were relatively fewer measurements then (e.g. only Syowa measurements are available for 1979–1984) and this period also includes the El Chichon aerosol induced ozone loss there (e.g. Figure S2, bottom panel). The resulting estimates (Fig. 2f) show a reduction in ozone trends by about 2–5%/year at 400–550 K in 1986–2001 and about 1–3%/year at 375–450 K in 2001–2013 compared to the standard scenario.

The trends estimated without using the regression terms, and employing linear trend terms only, provide a test of the influence of the regression uncertainties; this returns similar values to that of the standard scenario for both periods (Fig. 2g, “LINT”). The uncertainties of the trends are, however, larger at all altitudes and become insignificant at the 95% level at 400 K. This is expected, since a simple linear trend analysis will not account for the changes in ozone due to effects of different chemical and dynamical processes. This motivates the use of the multi-linear regression method to identify chemical ozone trends.

The robustness of the positive trends in 2001–2013 and its altitude range are further tested with respect to different confidence levels and it is found that the trends are significant even at the 99% level from 350 K to 550 K (Fig. 2h, shaded area). Similar results were also obtained from the TCO measurements inside the vortex, for which the trends are significant at the 99.9% level (around +1.72 ± 1.36%/year or +3.68 ± 2.91 DU/year) over 2001–2013 for all cases (including those with or without the dynamical terms heat flux or AAO). This significance level did not alter when the 1991–1995 data were removed from the vortex-averaged analyses, though the analyses without considering the vortex are significant at the 95% level only. The results from both ozone profile and column analyses suggest that the trends computed from the measurements with and without vortex criterion are not directly comparable. Nevertheless, these results (Fig. 2a–h) clearly indicate that the Antarctic ozone is recovering.

The robustness of the positive trends in 2001–2013 and its altitude range are further tested with respect to different inflection points from 1995 to 2002, in addition to the year 2000 shown in Fig. 2. It is found that the trends estimated for each scenario are significant at the 95% level from 350 K to 550 K (Figure S5, shaded area).

**Figure 3:**
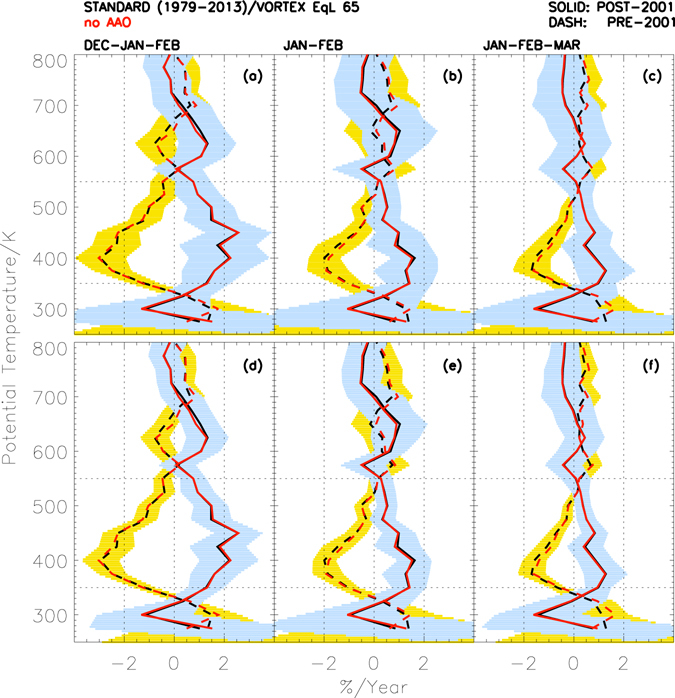
Vertical structure of ozone trends in Antarctic Summer: (**a**) Vortex averaged (≥65° S EqL) ozone trends estimated from ozonesonde measurements in Antarctica for (**a)** December–January–February (DJF), **(b)** January–February (JF) and **(c)** January–February–March (JFM) seasons. The shaded areas represent their significance at the 95% level (**a**–**c**, upper panel) and 85% level (**d**–**e**, lower panel). The red curves represent the analyses without considering the influence of the Antarctic Oscillation. Note that the number of observations in summer (DJF, JF and JFM) is significantly fewer than those in spring (SON, Fig. 2). In all plots (**a**–**f**) the horizontal dotted lines represent 350 K (~12 km) and 550 K (~22 km) altitudes and vertical dotted lines represent −2%/year and 2%/year.

To further examine the ozone recovery using the season with the minimum of dynamical influence, we next examine summer trends^16, 35, 36^, i.e. January, February and March (Fig. 3). The trends are estimated for three different averaging intervals; December–January–February (DJF), January–February (JF) and January–February–March (JFM). In DJF, the trends during 1979–2001 are about −0.5 to −2.5%/year at 350–550 K, with a peak around 400 K. The recovery trends during the period 2001–2013 show about 2–2.2%/year at 400–450 K, and about 1–1.5%/year below and above that altitude range. The trends in both periods show similar behavior to that of SON, although the values are about 5 times smaller in DJF. In JF, the trends in 1979–2001 are about −1 to −2%/year, while those in 2001–2013 are about 1–1.5%/year with the peak around 400 K in both periods. The trends are about 0.5%/year smaller in JFM in both periods and the peak values are shifted to lower altitudes of about 375 K (~12 km), as may be expected due to slow descent through the season. The estimated trends are significant at the 95% level in DJF at 350–520 K (~11–21 km), 350–420 K (~11–15 km) and 460 K (~17 km) in JF, and 350–370 K (~11–12 km) and 470 K (around 18 km) in JFM. The results did not change significantly when the AAO influence was not considered in the regression (see the red overlaid curves in Fig. 3). Overall, the trends are significant at the 85% level for all seasons at 350–520 K (Fig. 3, lower panel).

The TCO measurements in summer (DJF) further attest to the recovery displayed in the ozonesonde profile observations, as the analysis yields positive trends of about +0.51 ± 0.47%/year (+1.50 ± 1.39 DU/year) in summer for the 2001–2013 period. The trends are significant at the 90% level and they remain significant at the same level even when the dynamical effect of AAO is subtracted (i.e. +0.59 ± 0.50%/year or +1.74 ± 1.46 DU/year), consistent with the results obtained from the ozone profile measurements. These results strongly support the identification of the onset of ozone recovery, in tandem with the chemistry and dynamics in spring.

In closing, we note that while transient ups and downs are to be expected from one year to another, the behaviour of Antarctic ozone trends over the longer term since 2000 reveals clear signs of recovery. Our results robustly suggest that the successful implementation of the Montreal Protocol to protect stratospheric ozone has begun to save the Antarctic ozone hole.

## Methods

We have used ozonesonde measurements from nine stations in Antarctica (as available from the World Ozone and UV data center [WOUDC] and Network for the Detection of Atmospheric Composition Change [NDACC]) representing the south polar vortex region. The ozonesonde data used include those from Davis (68° S, 77° E; 2003–2012), Georg-Foster (70° S, 08° W; 1985–1992), Maitri (70° S, 11° E; 1994–2008), Marambio (64° S, 56° W; 1988–2013), McMurdo (77° S, 166° E; 1986–2010), Mirny (66° S, 93° E; 1989–1991), Neumayer (70° S, 08° W; 1992–2013), South Pole (90° S, 0° E; 1986–2013) and Syowa (69° S, 39° E; 1979–2013). A careful examination of the data is carried out by looking individually at each profile from each station. Suspected data with unreasonable values such as spurious spikes or erroneous balloon temperatures are exempted. The profiles terminating (bursting altitude) below 450 K (∼15 km) are also discarded. In addition, the correction factor either given in the ozonesonde metadata or calculated using the satellite measurements over the stations is applied to correct the individual ozone profiles. To calculate the missing correction factors we have used the overpass data from the Total Ozone Mapping Spectrometer (TOMS) in 1979–2003^37^ and the Ozone Monitoring Instrument (OMI) in 2004–2013^38^, where the missing years (1994–1995) are filled with the Multi Sensor Reanalysis (MSR) data^39^. The same TOMS and OMI data are used for the TCO trend calculations. Profiles with correction factors between 0.8 and 1.2 for the Electrochemical Concentration Cell (ECC) sondes^40, 41^, and those between 0.9 and 1.5 for the Brewer sondes are included in our analyses^42^.

The SON data are grouped inside the vortex by applying a criterion of EqLs ≥65° S at each altitude and averaged over all stations. We have used the Nash *et al*.^21^ criterion to compute the EqLs from the ERA-interim data^43^ and find negligible difference between the ozone data analysed using ≥65° S EqL and ≥70° S EqL criteria.

The heat flux data are computed from the ERA-interim meteorological analyses and details of the calculations are given in Kuttippurath *et al*.^25^. The EEASC data are obtained via NASA auto-mailer system. The solar flux (SF) data are from ftp://ftp.ngdc.noaa.gov/STP/SOLARDATA/SOLARRADIO/FLUX/PentictonAdjusted/monthly/, the quasi-biennial oscillation (QBO) data are from http://www.geo.fu-berlin.de/met/ag/strat/produkte/qbo/, and the aerosol optical thickness data are from http://data.giss.nasa.gov/modelforce/strataer/, the AAO data are from http://www.ncdc.noaa.gov/teleconnections/, the total column ozone data of TOMS are from http://toms.gsfc.nasa.gov/ozone/ OMI data are from http://avdc.gsfc.nasa.gov/pub/data/satellite/Aura/OMI/V03/L2OVP/OMTO3/, and MSR data are from http://www.temis.nl/index.php.

## Electronic supplementary material

SUPPLEMENTARY FILE
