# Supplementary material for: The signs of Antarctic ozone hole recovery
Source: Sci Rep. 2017 Apr 3;7:585. doi: 10.1038/s41598-017-00722-7 (PMC5429648; doi:10.1038/s41598-017-00722-7)
Supplement: Supplementary file 1 — SUPPLEMENTARY FILE [file 41598_2017_722_MOESM1_ESM.pdf]

The signs of Antarctic ozone hole recovery

Jayanarayanan Kuttippurath[1, 2]*and Prijitha J. Nair[1,3]

[1]*CORAL, Indian Institute of Technology Kharagpur, Kharagpur–721302, India.*

[2]*LATMOS/CNRS, UPMC University of Paris 06, Paris, France.*

[3]*Centre for Earth Science Studies, Thiruvananthapuram, India.*

** To whom correspondence should be addressed. Email: jayan@coral.iitkgp.ernet.in*

Running head: Antarctic ozone recovery

SUPPLEMENTARY MATERIAL / EXTENDED DATA

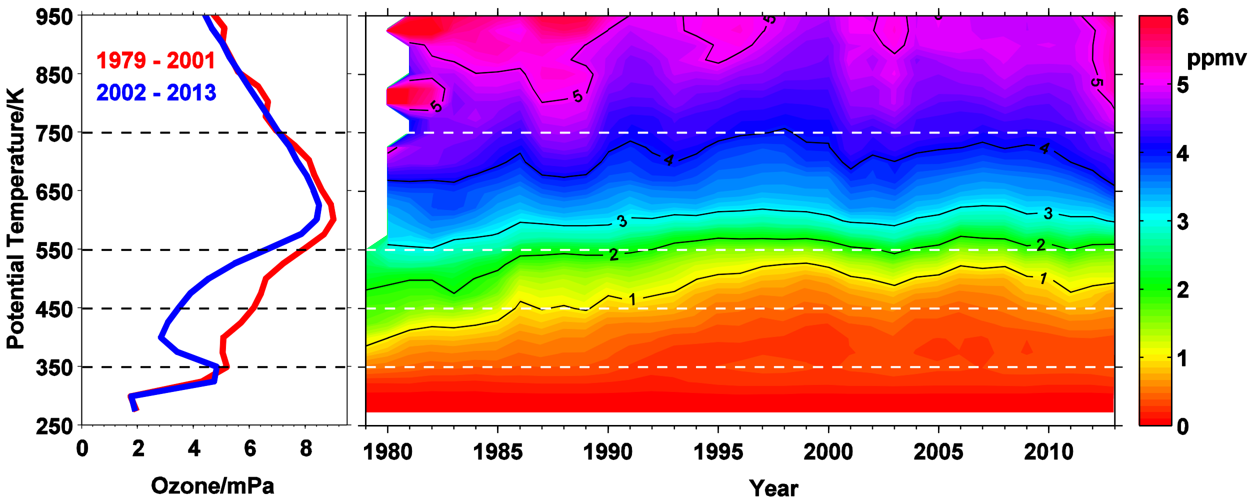

Figure S1. Evolution of Antarctic Ozone. **Left:** The vortex averaged (≥ 65°S EqL) ozone profile measurements (millipascals) from nine Antarctic stations averaged over September, October and November (SON, spring) before and after the stratospheric halogen maximum period (2001). The profiles show large ozone loss during the peak halogen period as compared to the 1979—2001 period. **Right**: The time evolution of vortex averaged ozone profiles (parts per million in volume) in the Antarctic from 1979 to 2013 in spring**.** The ozone time series also display a gradual increase in ozone in the lower stratosphere. The horizontal dashed lines represent 350 K (~12 km), 450 K (~16 km), 550 K (~22 km) and 750 K (~30 km) potential temperature levels.

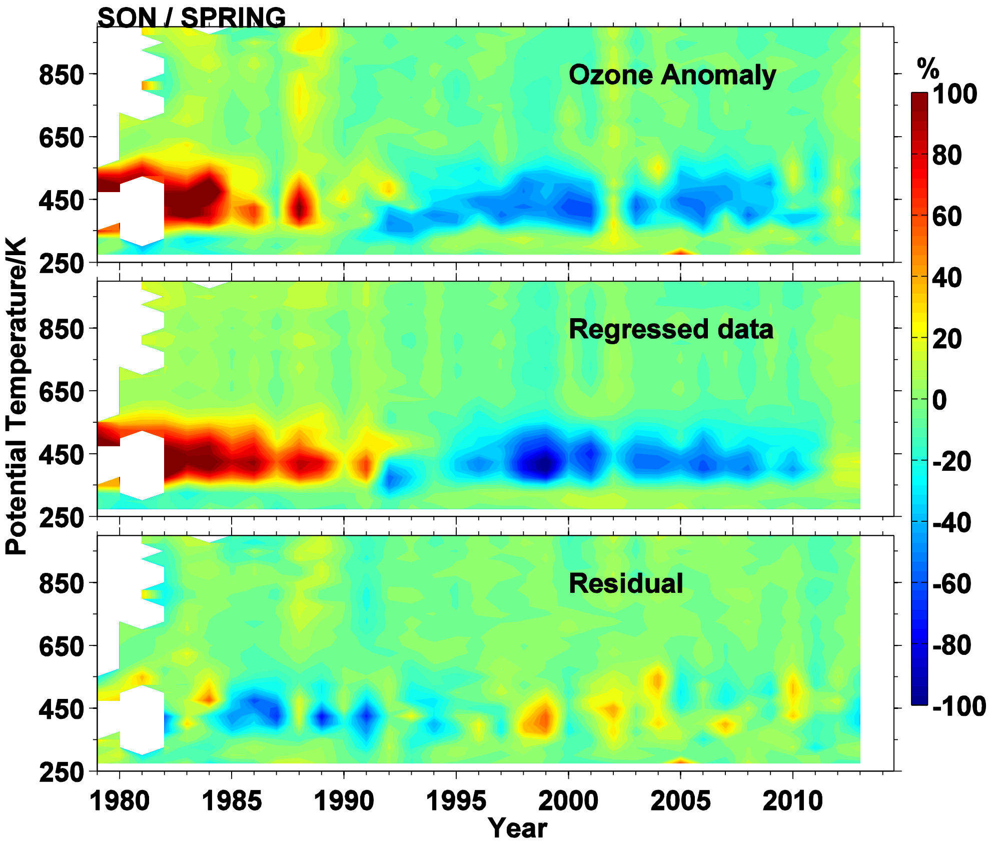

Figure S2. Regression analysis. The springtime (September–November) evolution of Antarctic ozone anomaly (shown in Figure 1). **Top to bottom**: Ozone Anomaly, Regressed data, and Residual.

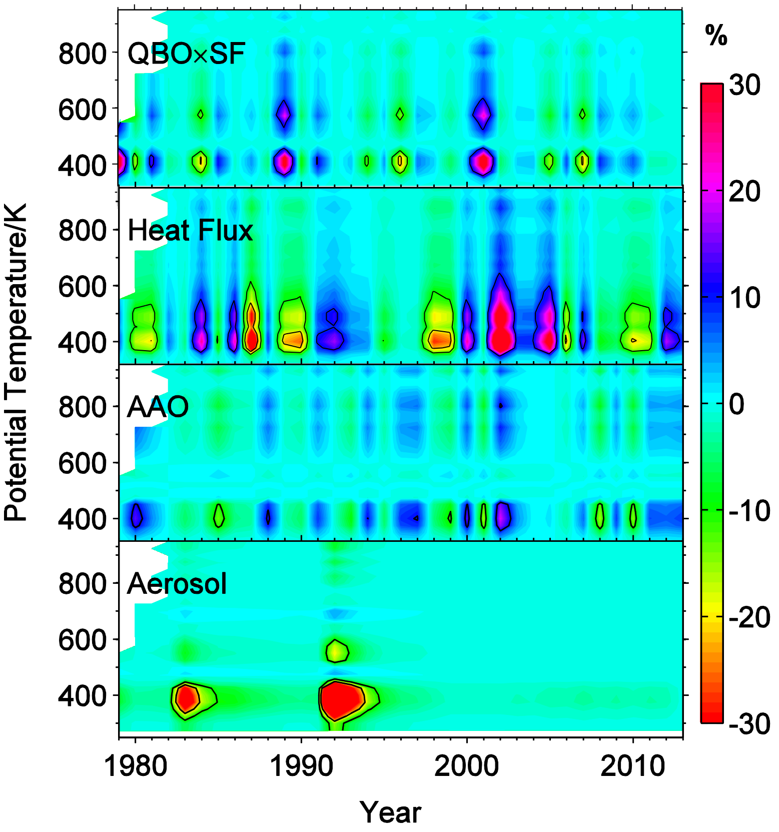

Figure S3. Drivers of ozone change. The springtime (September–November) evolution of the contributions of various proxies to Antarctic ozone (shown in Figure 1, Figure S2) change deduced from the piece-wise linear trend (PWLT) regression. **Top to bottom**: Quasi-biennial Oscillation (QBO) coupled with solar flux (SF), Heat flux, Antarctic Oscillation (AAO) and Aerosols.

Figure S3 shows that the heat flux, i.e. planetary wave driving of the mean circulation is the main source of ozone variability among the proxies and is predominantly influential at 350–550 K (~12–22 km). For instance, relatively large wave activity during 1986, 2002 and 2004 induced weaker vortices and contributed to a positive ozone change, while weak activity in 1982, 1987, 2000, 2006 and 2008 produced strong vortices and contributed to a negative ozone change, with an amplitude of about 20–30% in both cases[14]. The SF and

QBO contributions to ozone change are largest during the solar maxima and smallest during the solar minima years, with a peak value of about +20% in 1989 and 2001[14,27]. The contribution from the AAO depends on the meteorology of each year[25], as in the case of heat flux, e.g. with about +20% in 2002 and -10% in 1985. However, the change in ozone from the contribution of El Chichon and Mt. Pinatubo volcanic aerosols outweighs others[28,29] in 1982–1983 and 1991–1993 at 350–450 K, respectively, at about -30%.

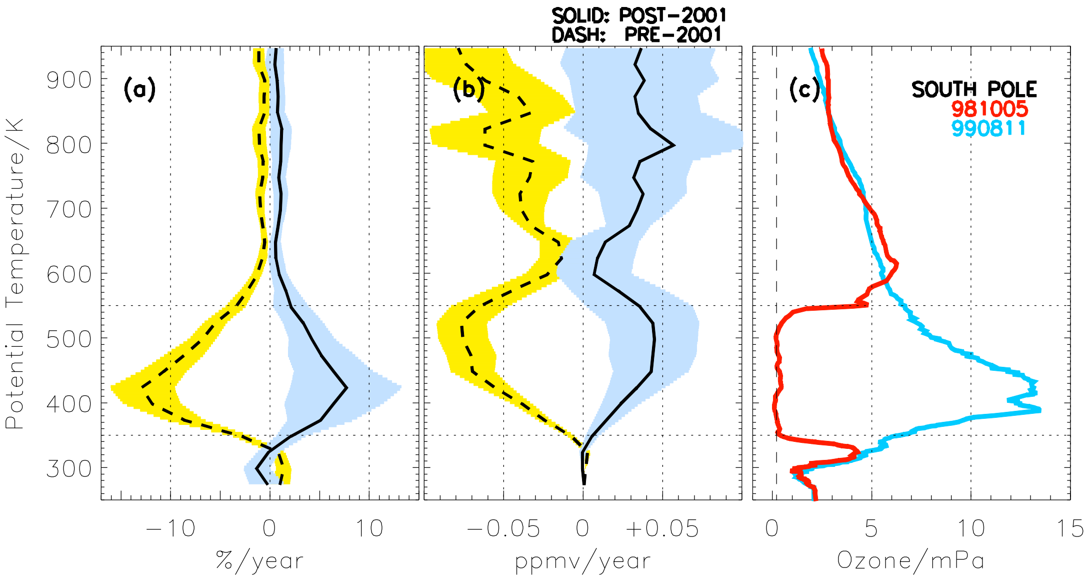

Figure S4. Vertical trends in ozone. **(a)** The vortex averaged (≥ 65° S EqL) springtime (September–November) ozone trends estimated from the Antarctic ozonesonde measurements using the piece-wise linear trend (PWLT) regression for the 1979–2001 (dash) and 2001–2013 (solid) periods. The shaded areas represent their significance at the 95% level. The break year 2001 corresponds to the year of maximum stratospheric chlorine in the Polar Regions. The vertical dotted lines represent +10 %/year and -10 %/year. **(b)** Same as (a), but in units of parts per million (ppm) per year. **(c)** A typical example of ozone loss saturation observed at South Pole (in millipascals or mPa), compared to a winter profile when there was little or no ozone loss. The vertical dotted lines represent 5 mPa and 10 mPa, and the dashed line represent 0.2 mPa, the detection limit of ozonesondes. The horizontal lines (**a–c**) represent 350 K (~12 km) and 550 K (~22 km) altitudes.

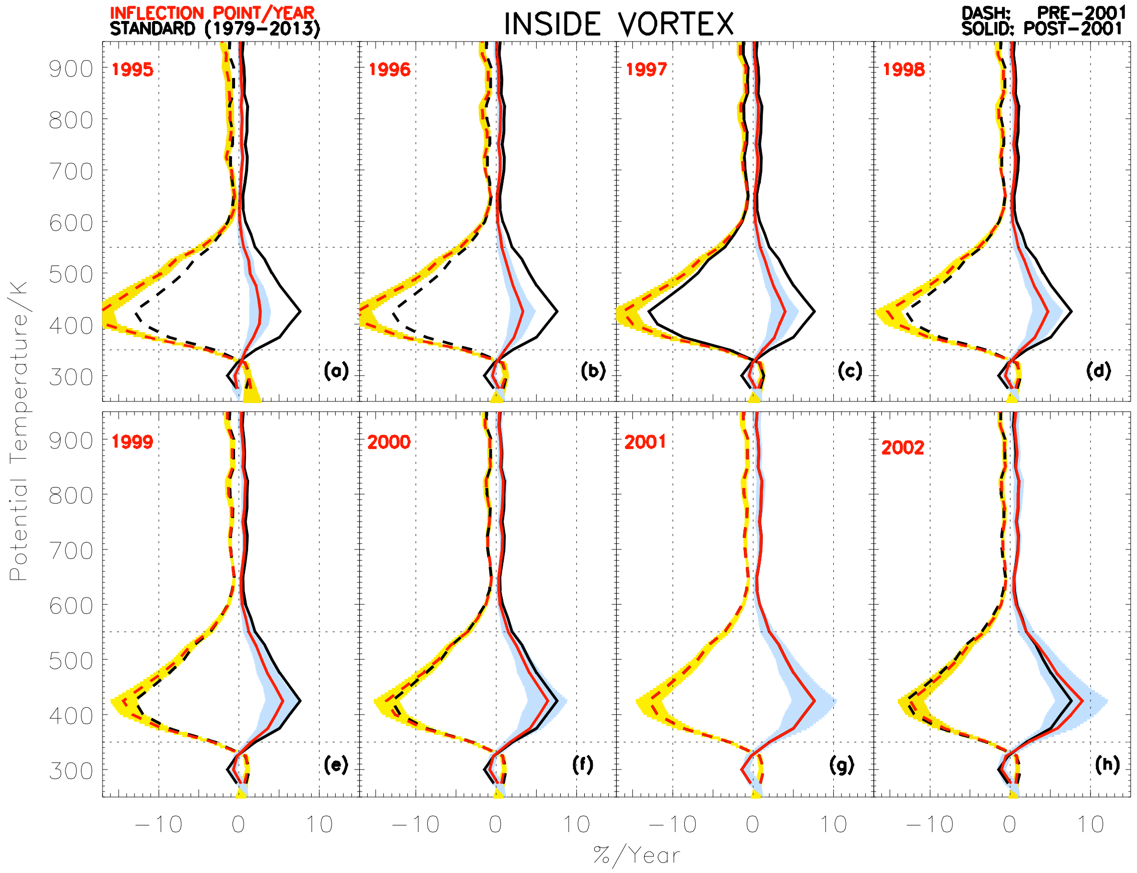

Figure S5: Sensitivity of Antarctic ozone recovery to inflection points**: (a):** The ozone trends estimated from ozonesonde data in the Antarctic vortex in spring (SON) with ≥ 65° S EqL criterion ("standard", black cures) for the 1979–2001 (dash) and 2001–2013 (solid) periods. The break year 2001 corresponds to the year of maximum stratospheric chlorine in the Polar Regions. The sensitivity tests with different inflection points from 1995 to 2002 are illustrated in red curves from (a) to (h). The shaded areas represent its uncertainty at the 95% level. In all plots (a–h) the horizontal dotted lines represent 350 K (~12 km) and 550 K (~22 km) altitudes and vertical dotted lines represent -10 %/year and 10 %/year.
